# Implementation of Common Rule Changes to the Informed Consent Form: A Research Staff and Institutional Review Board Collaboration

**DOI:** 10.31486/toj.19.0080

**Published:** 2020

**Authors:** Grace Gartel, Heather Scuderi, Christine Servay

**Affiliations:** ^1^ Kayentis, Boston, MA; ^2^ Ochsner Clinic Foundation Institutional Review Board and Clinical Trials Coordinator, Ochsner Clinic Foundation, New Orleans, LA; ^3^ Ochsner Clinic Foundation Institutional Review Board, Ochsner Clinic Foundation, New Orleans, LA

**Keywords:** *Consent forms*, *ethics committees−research*, *research personnel*, *research subjects*

## Abstract

**Background:** The Common Rule, which governs federally funded clinical research involving human subjects, formally defines the requirements for institutional review board (IRB) membership, functions and operations, and review of research, as well as the requirements for obtaining informed consent from research participants. The revisions to the Common Rule effective in January 2019 changed some content requirements for informed consent forms.

**Methods:** This article summarizes the history of informed consent requirements, the changes made to the requirements by the revision to the Common Rule, and the ways in which IRBs and research staff work together to develop informed consent forms that comply with the regulations and provide all the information potential research subjects need to decide whether to participate in a study.

**Results:** Clinical research coordinators, under their investigators’ supervision, are responsible for ensuring that research consent forms comply with the requirements of the federal regulations and the institution. Many IRBs have provided education regarding these new requirements, as well as consent templates that contain all the required elements. To ensure that the Common Rule's requirements are met, the IRB reviews each study submission, including the consent form. The IRB panel makes revisions to the consent forms as needed and returns the approved consent form to the investigator and clinical research coordinator.

**Conclusion:** Research coordinators play an essential role in developing consent forms and providing the required review information to the IRB. In turn, through optimizing and standardizing consent forms and ensuring that all requirements of the Common Rule are followed, IRBs ensure that the rights of participants are protected and upheld.

## INTRODUCTION

The basis for obtaining informed consent in medicine originated in US legal cases in which patients sued their surgeons for battery. The earliest cases (1905), *Mohr v Williams* and *Pratt v Davis*, established the legal precedent for a patient's right to be in control of decisions made about his/her medical treatment. The 1957 case *Salgo v Leland Stanford Jr. University Board of Trustees* was the first instance of the use of *informed consent* as a legal concept.^1^ Also key in the development of informed consent regulations were the passage of the Federal Food, Drug, and Cosmetic Act of 1938 that required human trials of new drugs to establish safety before they could be marketed to the public and the 1947 publication of the Nuremberg Code following the Nazi war crimes trials after World War II.^2-4^ The Nuremberg Code outlined the basic requirements of ethical human subjects research, including the need for informed consent.^3,4^ Published in 1964, the Declaration of Helsinki was the first international code of ethical principles regarding human experimentation, and in 1966, the US Surgeon General released a policy statement emphasizing the need for independent review of research projects before they could begin.^2-4^

However, serious ethical transgressions continued to occur, as was made clear in 1972, when an Associated Press story about the Tuskegee Study of Untreated Syphilis in the Negro Male, commonly known as the Tuskegee Study, caused public outrage about research misconduct.^5^ This study was designed to follow the natural progression of syphilis in the African American population and began in 1932. Participants were told that they would be treated for “bad blood,” a local term for a number of problems including syphilis and anemia, and participants agreed to be examined and treated. Although the study was initially planned to last 6 months, it continued for 40 years. An independent panel review determined that researchers had not informed Tuskegee participants that they were in a study, had not obtained informed consent, had not offered adequate treatment when it became available, and had not notified participants that they could withdraw and seek treatment elsewhere. The revelations about the Tuskegee Study were a significant contributor to the passage of the National Research Service Award Act of 1974 that required all research funded by the US Department of Health, Education, and Welfare (now the Department of Health and Human Services) to undergo institutional review board (IRB) review as a condition of the funding.^6^

The National Research Act of 1974 established the IRB to protect research participants’ rights and also created the National Commission for the Protection of Human Subjects of Biomedical and Behavioral Research that released the Belmont Report in 1979.^2,6,7^ The Belmont Report, which established the research ethical principles of respect for persons, beneficence, and justice, became the basis of the 1991 Federal Policy for Protection of Human Subjects (45 CFR §46)—known as the Common Rule—that governs “all research involving human subjects conducted, supported, or otherwise subject to regulation” by the signatory federal departments and agencies; defines the requirements for IRB membership, functions and operations, and review of research; and defines the requirements for obtaining informed consent from research participants.^2-4,8^

The 1991 Common Rule required the IRB to ensure that informed consent was obtained from research participants or their legally authorized representatives prior to involving them in a research project. The informed consent form and process must notify potential participants that the study involves research, provide an explanation of the research purposes and the expected duration of the study, describe the research procedures and identify any experimental procedures, and explain the risks and benefits and possible alternatives to enrolling in the study. The informed consent must also define the methods by which confidentiality of records will be maintained, state whether and how subjects will be compensated, inform participants of the availability of treatments if they are injured as a consequence of their participation, and provide contact information in case of questions or research-related injuries. Perhaps most important, informed consent also requires the statement that “participation is voluntary, [and] refusal to participate will involve no penalty or loss of benefits to which the subject is otherwise entitled, and the subject may discontinue participation at any time without penalty or loss of benefits.”^8^ In addition, the informed consent process has to include adequate time for potential participants to decide whether to participate and must “minimize the possibility of coercion or undue influence.”^8^

The Common Rule requires that all explanations given to potential participants be in understandable language and that no attempts be made to waive any of the subject's legal rights or to limit liability. Additional elements of consent that must be included when appropriate include information on (1) whether the treatment could involve unforeseen risks to the subject (or the subject's embryo or fetus), (2) whether and how subjects could be withdrawn without their consent, (3) any costs to subjects caused by their participation, (4) withdrawal and early termination procedures, (5) procedures for informing subjects of new information that could affect their willingness to continue in the study, and (6) the approximate number of participants planned for the study.^8^

## CONSENT FORM CHANGES

In 2017, the Common Rule was updated for the first time to strengthen protections for human research volunteers, reduce some of the regulatory burden on researchers and IRBs, and modernize the regulations in light of advances in technology and research practices. Most provisions of the rule became effective in 2019, and they include several changes to consent forms.^9-11^

### Key Information Section

Consent forms are long and complex, so they are not always easy for potential participants to understand.^12,13^ Potential participants may have difficulty understanding the information needed to make informed decisions.^12,14^ Ideally, language should be written at an 8th grade reading level or below,^15,16^ but this goal is not always achieved in practice.^14,15^ In a review published in the *New England Journal of Medicine*, the average reading level of consent forms was grade 10.6,^16^ and reading levels above the 8th grade level have been corroborated in other reports.^15^

One revision to the Common Rule is designed to improve participants’ comprehension of a proposed study, as important information has often been buried in the long, complex forms. Consent forms for federally funded studies must now include a “concise and focused presentation of the key information” at the beginning of the form in understandable language so that a reasonable lay person can evaluate whether to participate in a study.^9,17^

Because the US Food and Drug Administration (FDA) did not harmonize its regulations with the revised Common Rule implementation in 2019, this requirement does not currently apply to FDA-regulated trials. Until the FDA revises its regulations, including the key information section in consent forms for FDA-regulated trials is acceptable but not required.

This revised Common Rule allows flexibility in how the key information about the trial is presented. In 2018, the Office for Human Research Protections provided guidance at its website in anticipation of the new regulations.^17,18^ Even with this guidance, however, no consensus has been reached on the specific information to be included in the key information section. In December 2018, Le et al published the results of their survey of IRB members, investigators, and medical monitors asking about the information that should be included in the key information section of informed consent forms.^19^ Responses varied considerably. Of the 39 possible risks listed on the survey, all respondents identified 7 risks that should be included in the key information section. Some respondents preferred that most of the risks be included in the key information section, while others disagreed. Other papers report differences in opinion among potential participants and investigators concerning what information is important to convey in the consent forms.^20-22^

### Identifiable Private Information and Identifiable Biospecimens

New regulations also apply to identifiable private information and identifiable biospecimens (eg, blood, urine, or tissue samples) that are collected from participants in research trials. The consent form must state whether the information and specimens from the current research may be stripped of identifiable information and used for future research.^9,17^

Three additional elements for consent concerning biospecimens must be included under the new rule if they apply to the study.^9^ Individuals must be informed whether

- their biospecimens may be used for commercial profit
- the research may include whole genome sequencing
- clinically relevant research results, including individual results, will be disclosed, and if so, under what conditions

Research using biospecimens aids in the development of new drugs, devices, and diagnostic tests. Therefore, participants must understand that sponsors may profit from the use of their specimens. Whole genome sequencing involves determining an individual's complete DNA.^23^ Sequencing may be done to analyze mutations in malignant tumors to test mutation-specific treatments or to identify hereditary disorders. Some potential participants may have concerns that whole genome sequencing could have negative consequences. Finally, sometimes research reveals medical information that participants otherwise would not know. For example, tests performed at a central research laboratory could suggest that a participant has diabetes mellitus or human immunodeficiency virus. Potential participants need to know if they will be informed of such clinically relevant results. This knowledge could affect a participant's medical care and/or willingness to continue participation.

## ROLES OF THE INSTITUTIONAL REVIEW BOARD AND RESEARCH COORDINATORS

While implementation of the new Common Rule regulations is not required for trials that are not federally funded, implementing the new requirements is considered best practice.^24^ However, implementation varies among IRBs. Some IRBs at institutions, such as the Ochsner IRB, are strictly following the new rule and require the key information section only for federally funded studies.^25^ Some IRBs recommend or require a key information section for consent forms unless they are very simple and short.^26,27^ Some institutions now require that all consent forms for research trials include the key information section.^24^ Many IRBs at institutions and private companies provide training and/or guidance information on their websites to explain and implement these new rules.^24,25,27-30^

Because the Common Rule allows flexibility in how the key information section is written, an IRB-prepared consent template is a great aid to the research staff. Many IRBs require that investigators use the institution's consent template.^25,27,28,31,32^ These templates include any specific language required by the state and the institution. Many IRBs have revised their templates to include the new Common Rule information.^24-26,29,31^

Clinical trials sponsors typically provide a model consent form along with the study protocol. Typically, a research coordinator transfers the sponsor's consent language to the IRB consent template used at the institution.^25^ Care must be taken to avoid redundancy, as the sponsor's language often contains the same elements of consent as the template but worded differently.

Some IRBs provide a lay term glossary to help research staff simplify the medical terms used in consent forms.^25,32^ IRBs often have consent writing guidance on their websites and/or provide in-person training.^24,25,29,31^ Research staff may also seek advice from IRB staff about specific consent forms prior to submission.

### Institutional Review Board Submission and Review

The research coordinator helps the investigator submit all pertinent research documents to the IRB for review, including the protocol, consent forms, advertising information, and study drug and/or device information.

At Ochsner, the IRB staff reviews each submission to ensure that it meets all of the basic requirements.^25^ All members of the IRB panel are provided electronic access to the study documents for review prior to the meeting. All information provided to potential subjects, including brochures, videos, social media posts, posters, and advertising, is considered part of the consent process and is reviewed. Each study is assigned a primary study reviewer and primary consent reviewer.^25^

The consent reviewer assesses the informed consent document to ensure that all required elements of the consent are present and redundancies are removed, the document is in simple lay language, all procedures in the consent form match the procedures listed in the protocol and are explained in detail when necessary, and risks listed in the consent form are consistent with the protocol and the drug/device information submitted.^25^ If the consent reviewer has questions, the reviewer can contact the IRB staff or the research coordinator to resolve the issues prior to the IRB panel meeting.

The investigator, often with the research coordinator present, typically presents the study to the IRB panel in person or by telephone and answers questions about the protocol and/or consent form.^25^ After the investigator leaves, the panel members discuss the study and the consent form. Various panel members, including physicians and nonscientists, provide input, and the consent form is amended as needed. The IRB-approved consent form is returned to the investigator and coordinator for submission to the sponsor.^25^ Other institutions and IRBs follow similar procedures for reviewing clinical trials and approving consent forms.^26-28,31,33^

## CONCLUSION

A supportive relationship between research coordinators and IRB members helps ensure that consent forms are legally compliant and ethically sound. Research coordinators play an essential role in developing consent forms and providing the required review information to the IRB. In turn, through optimizing and standardizing consent forms and ensuring that all requirements of the Common Rule are followed, IRBs ensure that the rights of participants are protected and upheld.

## Notes

This article meets the Accreditation Council for Graduate Medical Education and the American Board of Medical Specialties Maintenance of Certification competencies for Patient Care, Medical Knowledge, and Systems-Based Practice.
